# Paediatric early warning systems: not a simple answer to a complex question

**DOI:** 10.1136/archdischild-2022-323951

**Published:** 2022-07-22

**Authors:** Damian Roland, Colin Powell, Amy Lloyd, Robert Trubey, Lyvonne Tume, Gerri Sefton, Chao Huang, Katie Taiyari, Heather Strange, Nina Jacob, Emma Thomas-Jones, Kerenza Hood, Davina Allen

**Affiliations:** 1 SAPPHIRE Group, Health Sciences, University of Leicester, Leicester, UK; 2 Paediatric Emergency Medicine Leicester Academic (PEMLA) Group, University Hospitals of Leicester NHS Trust, Leicester, UK; 3 Department of Emergency Medicine, Sidra Medical and Research Center, Doha, Qatar; 4 Division of Population Medicine, Cardiff University, Cardiff, UK; 5 Centre for Trials Research, Cardiff University, Cardiff, UK; 6 School of Health and Society, University of Salford, Salford, Greater Manchester, UK; 7 Alder Hey Children's NHS Foundation Trust, Liverpool, Merseyside, UK; 8 Hull-York Medical School, University of Hull, Hull, UK; 9 School of Healthcare Sciences, Cardiff University Centre for Trials Research, Cardiff, Wales, UK

**Keywords:** child health services, emergency service, hospital, health services research, mortality, paediatrics

## Abstract

Paediatric early warning systems (PEWS) to reduce in-hospital mortality have been a laudable endeavour. Evaluation of their impact has rarely examined the internal validity of the components of PEWS in achieving desired outcomes. We highlight the assumptions made regarding the mode of action of PEWS and, as PEWS become more commonplace, this paper asks whether we really understand their function, process and outcome.

## Background

In adult clinical practice, the incidence of preventable inpatient mortality is significant (with potentially over 22 000 preventable deaths annually in the 1USA) and has precipitated the development of a variety of interventions to standardise the processes for recognition and response to evolving inpatient deterioration. These have been delivered by the use of early warning systems (EWS) to aid recognition and response to patient deterioration^2^ which have included:

1. The development of track and trigger tools^3^ (numerical values assigned to commonly measured physiological and observation values which produce a composite score that correlates to an escalating response process dependant on the score)
2. Rapid response teams (RRTs)^4^ (experienced staff assigned to respond to patients who trigger criteria predictive of impending need for intensive care) and
3. Critical care inreach and outreach^5^ (deploying specialist critical care staff to non-intensive care settings to prevent admission and outreaching to prevent readmission to critical care).

Determining effectiveness is challenging and data on system-level improvement in mortality are sparse. The concept of EWS in the UK was discussed as early as 1997^6^ and the Royal College of Physicians recommended their use in 2007.^7^ Since this point, publicly available data have shown inpatient hospital mortality has actually risen from 275 000 in 2014^8^ to 293 000^9^ in 2019 (uncontrolled for population growth or number of hospitals). The interventions (1–3) above have different impacts and the relationship between a specific intervention and an outcome is not always clear. For example an RRT is dependent on processes which enable not only initial recognition of deterioration, but require an institution to have experienced personnel available to make time-critical decisions on patients and have the capacity to provide appropriate resources if care needs to be escalated. It has been suggested that adult RRTs were implemented to address a problem without fully understanding the context of the problem and the system in which these problems exist^10^ but the face validity that RRT should work and the impetus to improve patient safety was so strong that limited evaluation of its mechanism of action took place. A subsequent systematic review has demonstrated a variety of non-patient-based factors to also be important,^11^ such as leadership and punitive hierarchies. This is similar to the introduction of pulse oximetry, which had minimal level 1 evidence supporting its introduction. The ease of measuring oxygen levels is so great it would now be unethical to undertake a study randomising patients to a control group.^12^ Acknowledging these experiences in adult practice by examining the link between intervention and outcome with a paediatric lens, we will explore what we know about the effectiveness of EWS for use in children (paediatric early warning systems, PEWS) and what this means for the development of outcome measures.

## Challenges with determining effectiveness

The first published Paediatric Early Warning Score in the UK was in 2005 with multiple iterations published since and nearly all hospitals in England now using some form of a score.^13^ In a recent systematic review, our group examined 30 studies to determine the effectiveness of paediatric track and trigger tools (PTTT).^14^ These studies were predominantly before and after studies using a variety of outcomes. These can be categorised into discrete outcome measures which have been grouped by frequency in table 1. There is great variation among studies in the precise definition of a code blue, cardiac arrest, respiratory arrest, unplanned admission to paediatric intensive care unit (PICU), and the criteria for PICU admission or high-dependency admission, making comparisons of PTTTs difficult. Denominator variation for mortality and cardiac arrest figures, different types of controls used in studies or indeed no control groups, as well as study design variability, all compound that challenge. In particular, denominators are challenging to calculate as they are often recorded as patient bed days, rather than raw patient numbers which can suffer from poor data recording (eg, over weekends).

**Table 1:** Range of outcome measures in a review of track and trigger systems^14^

Outcome measure	Number of studies
Cardiac arrest or code blue call	6
Urgent or unplanned PICU admission	17
Composite of death or unplanned PICU admission	15
Emergency medical intervention	2
Unplanned high dependency unit	2
Other	2

It is also important to note that most studies were conducted in specialist and tertiary centres where specialist intensive care advice is available on-site. These outcomes are therefore not representative of what is measured, or can be measured, in hospitals without direct access to intensive care. In these centres, where most hospitalised children in the UK are admitted, different decisions may be made about escalation, because of the lack of immediate access to both a PICU bed and critical care trained clinicians. Furthermore, children may deteriorate in one hospital without intensive care facilities but die in another which has them. It may then not be clear which interventions in either hospital were most impactful, or lacking, making causation and the role of PTTT difficult to ascertain. Therefore, if PTTT Scores are being used in non-specialist hospitals, it cannot be assumed the outcomes from their use will be the same as in tertiary hospitals. This also applies to their use in emergency departments, and prehospital settings, by paramedical staff and in primary care. PTTTs provide a mechanism to proactively highlight the need for review of a child who is becoming clinically unstable. In prehospital and emergency department locations a high PTTT Score may be representative of a child’s initial acuity rather than evidence of deterioration. The relationship between a high PTTT Score and a specific outcome is then less certain. Furthermore, the pretest probability for serious disease (which may result in deterioration) is much lower in emergency departments and prehospital settings therefore altering a PTTT’s specificity and sensitivity. It is perhaps for these reasons a paucity of evidence exists on the performance of PTTT Scores in these environments.^15 16^

## Challenges with delivering effective systems

The use of PTTTs with low specificity and low positive predictive values^17^ may result in alarm fatigue, short cuts to bypass the system and disengagement with the system overall.^18^ Adjustment of ‘normal’ vital sign triggering thresholds may be necessary for some high-risk groups of children (eg, children with congenital heart conditions), but if done by inexperienced staff or without evidence-based data, may reduce triggering of a PTTT and consequent failure to detect deterioration. Conversely, unnecessary critical care admission reduces capacity in an already strained system, is resource-intensive and may redirect experienced staff to clinical areas where they are not needed.

PEWS are complex due to the requirement for age-specific thresholds and difficulties ascertaining their effectiveness due to the lower mortality in children than in adults.^19^ All-cause mortality in children and young people continues to decline over time^20^ and a decade ago, Joffe and colleagues^21^ demonstrated that improvements in mortality previously attributed to patient safety systems, such as RRT, were also occurring in hospitals without such interventions. Childhood avoidable mortality in the UK (causes of death such as from infection or treatable conditions such as appendicitis) has fallen from 2726 in 2001, to 1902 in 2010 and then to 1473 in 2019.^22^ This trend infers that while PEWS have a role to play there are other systematic factors impacting mortality rates. Where mortality has been used as an outcome measure in evaluating the effectiveness of PTTT (and for comparison in adults there were 293 000 inpatient deaths in 2019) the studies are mixed,^23^ which has led to debate about their utility.^19^ A cluster randomised control trial comparing 10 hospitals in which the bedside PEWS (a PTTT) was implemented, with 11 control hospitals where there was no PTTT, failed to demonstrate an impact on mortality.^24^ During this study, that examined over 500 000 inpatient days in 150 000 children, there were fewer than two observed deaths per 1000 hospital discharges across both arms, and in approximately half of these deaths, ‘do not attempt resuscitation’ orders were in place. There has been one further randomised control trial published (comparing two different PTTT Scores) which concluded the results should be interpreted with caution given the low rate of clinical deterioration (only 22 unplanned transfers of care in 31 337 admissions to the recruiting paediatric hospitals).^25^ Therefore, it is reasonable to conclude the use of mortality as the primary outcome measure in these studies of PTTTs effectiveness is not robust.^26^

## Which are the most relevant outcomes?

While mortality is infrequent, it is catastrophic for both families and staff, so it is important we understand how these interventions may impact on the variety of processes that may lead to this tragic outcome. PEWS are a complex healthcare intervention,^27^ and it is difficult to determine which components impact on the quality of clinical processes, and the mechanisms by which each part exerts its effects. National reports continue to highlight the need for quality improvement in recognising and responding to inpatient deterioration and therefore healthcare organisations and regulators need to be able to assess which components of PEWS are effective across a range of process and outcome measures. Due to the focus on mortality as the end point in the deterioration pathway, some safety mechanisms that work well before deterioration and therefore avoid intensive care, may have been missed. There is a paucity of early or intermediate outcomes. Where they have been developed, they are rarely used as primary outcome measures.^28 29^ It is likely directing all improvement efforts to solely preventing mortality (as has legitimately occurred in adult practice) may impact on a host of balancing measures which affect productivity and resources.

Our recent evidence-based, theoretically informed, Paediatric early warning system - Utilisation and Mortality Avoidance. improvement programme (PUMA Programme) was developed and implemented in two general hospitals (no onsite PICU) and two tertiary hospitals (with onsite PICU) in the UK.^30^ We developed a composite metric (adverse events) as a primary quantitative outcome representing the number of children monthly that experienced one of the following: *mortality, cardiac arrest, respiratory arrest, unplanned admission to PICU or unplanned admission to a higher dependency unit*. Despite implementation challenges, all made contextually appropriate system changes with a decline in the adverse event rate at three sites. At the site in which system changes were organisationally adopted, this decline was significant. The variable impact on adverse rates highlights the dynamic qualities of PEWS. As an example, the introduction of an electronic EWS at one of the sites strengthened medical access to patient data but disrupted nursing work as there were insufficient computers available to allow nurses to enter vital signs, leading to a delay between monitoring and recording the PTTT Scores.

## How might PEWS function?

The lack of system-wide data demonstrating a specific impact of PEWS on mortality does not mean they are not effective for other outcomes, but that these outcomes have yet to be measured or realised. Furthermore, numerous different processes might contribute to an overall benefit, but a given individual intervention might not reach significance. These interventions may be relatively simple, from the production of minimum training standards for staff who will be involved in caring for patients on PEWS pathways, to complex electronic whiteboard systems which can highlight deterioration of patients, to command control centres remote from wards. Some interventions may also be embedded in processes which already occur, such as a morning handover. These may not previously have been regarded as interventions but will probably have been recognised as being beneficial by staff and institutions. Another example of an unrecognised intervention is that of situational awareness. This encompasses processes such as bringing staff together in huddles^31^ and identifying ‘watcher’ patients through shared communication processes.^32^ Watcher patients are those identified through nursing staffs’ tacit knowledge; for example, the identification of a patient who appears well at present, but the nurse’s experience dictates they are at risk of deterioration, even though the nurse cannot completely explain exactly why. Understanding whether, and more importantly how, these interventions take place needs a system assessment requiring more of a focused evaluation than a simple ‘yes/no’ questionnaire. A clinical area may claim they don’t use watchers, but this may be what staff are doing; conversely both clinicians and operational managers may claim they have strong situational awareness, but in reality, might be dependent on traditional communication hierarchies. Finally, it is also important there are the right number of staff, with the right tools, to enable them to perform the right task, at the right time. The availability (or absence of) equipment to effectively monitor a patient (such as functional pulse oximeters and correct sized blood pressure cuffs, etc) clearly impacts on the ability to detect and plan for a child becoming unexpectedly unwell. Adequate staffing is an important safety factor in itself and demand for child health services outstrips capacity with workforce issues being *‘a significant challenge to child health service delivery and improvement’* according to the latest Royal Collage of Paediatrics and Child Health review of the medical and nursing workforce.^33^

Interventions which aid parents’, carers’ and professionals’ ability to raise concerns about imminent deterioration in a child, and processes to measure this, would also provide an organisation with knowledge of this important facet of care. Organisational cultures have a significant impact on outcomes^34^ and actively demonstrating that patients, families and carers are involved in, and feel able to share concerns around, decision making is an important outcome in itself. Discrete outcomes such as mortality or ICU admission are easy to measure but other interventions, such as improving and maintaining situational awareness at a patient, ward and organisational level are also likely to have a positive impact on deterioration. They may also improve negative hierarchical cultures (an often unexplored but significant influencer on care) and therefore are an important part of clinical practice. The absence of studies examining the link between these organisational and human factor characteristics and specific outcomes is something that needs to be addressed with further study. A scoping review of PEWS undertaken in 2021 reiterated the need to understand both the ‘technical’ nature of any score and the wider social, cultural and organisational context in which they are deployed.^35^

Our PUMA programme assessment tool evaluates a system’s ability to Detect, Plan and Act in response to the deteriorating child. It provides a detailed description of what needs to be in place for children’s deterioration to be detected and acted on. This propositional model detailed in table 2 contains interventions, tasks and skill sets that are not always possible to link to specific patient outcomes but represent a positive safety culture in a particular ward environment. Constructs such as a no blame environment, empowerment, communication, situational awareness, psychological safety, clear leadership, closed loop feedback, teaching and so on are persistently recognised as being of importance. Certainly there is evidence from our qualitative examination of these systems that PEWS aid staff in having a common language for communication about deterioration.^36^ This might explain why PEWS have continued to spread^13 37^ despite equipoise on their utility and is a reason why national scores exist in Scotland and Ireland. The English National Health Service has started a schedule of activity to introduce a standardised inpatient PEWS chart.^38^ This work is part of an overarching System-wide Paediatric Observation Tracking (SPOT) programme. The utilisation of standardised outcomes and end points, being developed as part of the programme, will hopefully avoid further heterogeneous results which will be difficult to interpret. An expert working group has taken a list of potential outcome measures derived from a previous systematic review^14^ (box 1) and refined it to create a panel which will be measured in real time, both nationally and regionally, as the inpatient implementation rolls out. Following feasibility and usability exercises undertaken in a small group of hospitals a prototype chart has been rolled out in a number of pilot sites. This prototype chart has been developed via consensus opinion from multiple specialties with medical and nursing inputs. A community of practice has been created with weekly meetings examining enablers and barriers to effective de-implementation (all pilot sites already have a PEWS system in place and so this exercise is in changing rather than generating new practice). The pilot chart is continually being refined as a result of feedback from the pilot sites and a version 4.0 will be released in the late summer of 2022. The new version will undergo further real-world testing before a final version will be released later in 2022 for early adopter sites to implement. This programme of work has the support of National Health Service England (NHS England), The Royal College of Paediatrics and Child Health and the Royal College of Nursing with further information available via the Royal College of Paediatrics and Child Health (RCPCH) website.^39^

**Table 2:** The core components of a paediatric early warning system: the PUMA standard

	Proposition	Conceptual requirements
Detection	Detection of deterioration depends on timely and appropriate **monitoring** of vital signs and relevant risk factors	At a minimum, this requires: Staff are aware of which vital signs need to be monitored Staff are aware of the minimum frequency of observations required for the children in their care Staff are aware of the need to review the frequency of observations for children in their care Staff are aware of additional clinical assessments required for children with prior risk factors Monitoring tasks are allocated to staff members with appropriate skills to conduct them Staff have access to appropriate equipment to accurately monitor vital signs, and conduct other clinical assessments Staff are aware of roles and responsibilities for monitoring Staff have time to conduct accurate timely and appropriate monitoring of vital signs, alongside other work commitments Staff concern is formally recognised as a valid indicator of deterioration Staff are supported to develop and use their intuition in detecting signs of deterioration Staff understand the value of family concerns in the detection of deterioration Families are involved with defining normal physiological parameters for their child Families receive guidance about what to do if they are concerned that their child’s condition is deteriorating Staff keep families informed about developments in their child’s care and treatment
	Detection of deterioration depends on timely and appropriate **recording** of signs of deterioration	At a minimum this requires: Staff are aware of the need to record vital signs, family concern and staff concern promptly and accurately Staff are aware of roles and responsibilities for recording vital signs, family concern and staff concern Staff have appropriate skills to accurately record vital signs, family concern and staff concern Staff have access to appropriate equipment to accurately record vital signs, family concern and staff concern There are an appropriate number of staff to carry out required tasks
	Detection of deterioration depends on timely and appropriate **interpretation** of signs of deterioration	At a minimum this requires: Staff are aware of prior factors that increase children’s risk of deterioration (eg, premature birth) Staff are aware of roles and responsibilities for interpreting signs of deterioration Staff take into account vital signs, family concern and staff concern in assessing the condition of children in their care Teams have appropriate skills to discern patterns and trends of signs and symptoms Staff have the opportunity to learn how to interpret signs of deterioration from shadowing more senior staff Care is organised to enable staff to recognise patterns and trends for children Families are in a position to discern patterns of signs and symptoms in their child
	**Proposition**	**Conceptual requirements**
Planning	Planning depends on **reviewing** indicators of deterioration for each patient	At a minimum this requires: For each child, all indicators of deterioration are brought together and kept up to date There is a regular mechanism for reviewing the status of all children in the ward to identify those children who are a concern The is a regular mechanism for reviewing staffing levels and skills mix, workload, acuity and admissions
	Planning depends on staff being aware at ward level of the status of individual patients and the availability of skills and resources, and **preparing** an appropriate response	At a minimum this requires: There is a regular mechanism for communicating the review of all children, staffing levels and other resources to the rest of the team and senior managers There is a regular mechanism for planning appropriate response to deterioration Senior staff members are allocated responsibility for managing demand and resources Senior staff members are allocated responsibility for communicating response plans There is an action plan for children at risk of deterioration which is shared with families and staff caring for them
	**Proposition**	**Conceptual requirements**
Action	Action depends on clear **escalation** and response processes	At a minimum this requires: A trigger or prompt to act from detection or planning phases Clearly defined graded escalation and response procedures—agreed at organisational level Staff receive guidance about how to escalate and respond Staff understand their roles and responsibilities in the escalation procedure as activators and responders Staff are encouraged and supported in raising concerns Families are encouraged and supported in raising concerns Staff are able to communicate information across professional hierarchies using a structured approach to sharing information Clear structures to support action, including the use of a ‘no false alarms’ policy so staff are not deterred from escalating care
	Action depends on **evaluation**	At a minimum this requires: Escalation and response processes are reviewed to promote learning There is opportunity for staff to discuss differences of opinion in the need for escalation No blame is assigned to those who escalate

Box 1 **Outcomes used in all the paediatric early warning systems (PEWS) ‘validation studies’**

**Death**

1. Hospital-wide deaths per 100 discharges
2. Hospital-wide deaths per 1000 discharges
3. Ward deaths only per 1000 admissions
4. All-cause hospital-wide mortality rate
5. Deaths on paediatric intensive care unit (PICU)
6. PICU deaths for all ward transfers
7. PICU mortality rates among readmitted patients in 24 hours

**Cardiac arrests/respiratory arrest**

1. Near cardiopulmonary arrests
2. Cardiac arrest
3. Actual cardiopulmonary arrests
4. Unexpected cardiac arrests
5. Ward respiratory arrests per 1000 patient days
6. Preventable cardiac arrests

**PICU/PHDU**

1. Unplanned transfer to PICU (±within 24 hours of admission)
2. Unplanned PICU transfers per 1000 patient days
3. Critical deterioration events (life-sustaining interventions administered within 12 hours of PICU admission)
4. Invasive ventilation given to emergency admissions to PICU postintervention
5. Early intubation
6. Postintervention rates of PICU admissions receiving mechanical ventilation
7. Unplanned transfer to Paediatric High Dependancy Unit (PHDU) ±within 24 hours of admission)

**Urgent consult/review assistance/urgent review**

1. Code calls
2. Urgent calls to inhouse paediatrician
3. Urgent calls to respiratory therapist
4. Rapid response team (RRT) call
5. Outreach team calls
6. (Phone call advice/consults to PICU team)
7. Specific ‘Intervention’ with RRT—on ward

**Composite measures**

1. Combined cardiac and pulmonary arrests
2. Critical deterioration index (non-invasive or invasive ventilation and/or inotropic support within 12 hours after admission)
3. Critical deterioration index (non-invasive or invasive ventilation and/or inotropic support within 24 hours of admission)

## Conclusion

Healthcare organisations, academic institutions and regulators recommending PEWS as an answer to the problem of inpatient deterioration without understanding all the mechanisms of their action will deliver research and implementation studies with equivocal findings. The rapid spread of PEWS over the last decade is testament to their positive face validity and undoubtedly they have contributed to an improved safety culture. Ultimately we have not yet identified the specific ingredients and mechanisms by which PEWS work as we don’t have the outcomes by which to test different recipes. To improve outcomes for children in inhospital settings we must change the emphasis from rare outcomes (mortality) and examine and measure a wide range of interventions that may aid early detection of deterioration.

This work has arisen from a study/project funded by the National Institute for Health Research, Health Services and Delivery Research Programme, funder reference: 12/178/17. The views expressed are those of the author(s) and not necessarily those of the National Institute for Health Research or the Department of Health and Social Care.
